# On the Hygeine of Crime
*Crime: its Amount, Causes, and Remedies. By Frederick Hill, Barrister-at-Law, late Inspector of Prisons. 1 vol. 8vo. J. Murray, 1853.


**Published:** 1854-01-01

**Authors:** 


### Art. III.—ON THE HYGEINE OF CRIME.*

If the human race had obeyed the divine mandate, "Fear God, and love thy neighbour as thyself," crime would be an anomaly, and there would have existed no necessity for Mr. Hill to have written the interesting book now under review; for interesting it is, not merely because it displays, with something like a Rembrandt shading, the real night-side of human action, but because it inspires us with a hope that the adoption of the principles inculcated in its pages will lead to a diminution of crime, and an alteration in the treatment of criminals.

The criminal code is, of course, based on the infraction of those injunctions that were engraved on the sacred tables of stone. All crimes are, of course, sins; but there are some which, as they are matters at issue directly between the Creator and His creature, do not affect society, and therefore are not catalogued among *national* delinquencies. By the infraction of one of these commandments, although they may desecrate the heart, still there is no outbreak to constitute *illegality.* Four of them, however—murder, adultery, theft, and perjury—essentially affect the welfare and happiness of society, and become at once the special objects of the criminal law. Beccaria measures crime by the amount of injury inflicted on society. Crime would, *primâ facie,* seem to come legitimately under the analysis of the lawyer, inasmuch as the criminal code forms a large portion of his

* Crime: its Amount, Causes, and Remedies. By Frederick Hill, Barrister-at-Law, late Inspector of Prisons. 1 vol. 8vo. J. Murray, 1853.

practical study. But, unhappily, the law has been so deeply engrossed with the punishment of a *committed* crime, that it has hitherto almost delegated the duty of prevention to others. It would, indeed, be a blessing if the system of prevention had been more successful, that it were now possible to sheathe the bloody sword of justice; but the wish, though standing prominent in every philanthropic mind, is but an utopian dream; the prevention of crime will ever fall far short of our hope, constituted as human nature and human society are at the present day.

And to whom is this Christian commission issued? The divine monitor we must, of course, regard as the spiritual indicator to happiness, and to heaven. But, alas! that we are bound to record it, the coldness of many of the professors of religion is too often satisfied with doling out their homilies at stated times, half-forgetting that the very creatures who most need their pious exhortation are those who intentionally and habitually stay away from the recognised temples devoted to the worship of God.

There are, of course, numberless instances where fools "who went to scoff, remained to pray." Doubtless, many a pious divine has been constantly blessed with proselytes, especially those who deem their personal visitations, at least, as essential as their pulpit oratory. But how constantly are the most devoted efforts thwarted by latent influences, which the divine and the lawyer never dreamed of. How often has the scattering of good seed utterly failed because it has fallen on barren ground; or, what is as bad, a soil vitiated by the weeds of disease—and how often has the criminal, in consequence of diseased organization, lapsed again into his degrading courses, when his professions, perhaps his half-purified *intentions,* had promised better things.

In analyzing the able treatise before us, we may at first, perchance, alarm the timid spiritualist, by affirming, that in the great majority of even premeditated crimes, some morbid change has previously occurred in the organ of thought, or in those with which it intimately sympathizes. Let not the sensitive heart be scared by this affirmation—not for one moment would we assert this physical change to constitute any irresistible stimulus to crime. This would at once arrogate the doctrine of necessity, and remove all responsibility from the free agency of man. We merely mean to express our belief that deranged conditions of the body may influence the mind by sympathy as well as immediate disease of brain; and this may often suggest an extenuating plea for even grave offences against the person or the state. Indeed, the law itself has ever recognised this truth, that where there is a *mens insana,* which we know not to be a mere *metaphysical* state, it withholds the capital punishment for crime, and *detains* until the sovereign's pleasure decrees otherwise.

We perceive at once how deeply important to the other learned moralists is the science of the physician, in the matter of crime. His efforts are often essential—indispensable, indeed, in preparing the cerebral soil for the husbandry of the divine: and how constantly his experience is demanded in the court to enlighten the bar—ay, even the bench—with the light of pathology, when they would be else in dilemma as to the sanity and responsibility, or the madness and irresponsibility of an arraigned prisoner. If then the sages of the three learned professions would but join hands on the debatable ground of psychology, by such a union, we are certain, a world of blessing would be conferred on mankind. But the divine has been long wont to regard as his especial province, rather the remote causes; while the recognition of the exciting causes or motives of crime, seems to be the especial subject of the judgment-seat. Thus the third, or proximate cause, is completely overlooked. We hope—nay, freely acknowledge—that the pulpit has its multiform blessings; it may even dispossess many an evil spirit, and the law may exalt its penal tortures to frighten the mammon or the Moloch out of man's heart; but how, if the evil spirit of *disease* be there, will not that be a stumbling-block in their way? In such dilemma, they must come to the physician, to eradicate first the real poison from man's blood, or they may continue to preach or threaten in vain. We believe that such a blending of forces, if wisely effected, might even lighten the heavy weight of the Newgate Calendar, and prove a court of ease to the Old Bailey.

In treating this comprehensive work psychologically, we hope to go still further, and to show that, by ensuring a *corpus sanum*, we have the best chance of forming, by education and other trainings, a *mens sana;* and if these happy elements are in us, and abound, there will be even less dropping of black caps on judicial wigs, and far less of degrading iniquity in the common room of Newgate; and, what to the purse-bearer is of little less weight, a wondrous diminution of the county-rate.

We do not read, however, even the title-page of our author, whose office offered him the very widest field of observation, without noticing how nosologically he has arranged his subject: "amount," "causes," "remedy," are but more legal, or more popular, terms for *pathology*, *etiology*, and *treatment*,—and if we analyze further, we see, in truth, that the elements of every chapter are, probably without the consciousness of the author, psychological. The prevention of crime refers as much to the inculcation of good precepts and the withdrawal from bad example, as to the influence of bolts, bars, and scourges. Now, it would indicate very little influence on the mind, either by a precept or a fetter, were the amount of crime to remain in *statu quo;* yet the letter of Mr. Dufton to Lord John Russell, written ten years ago on this point, is not very flattering. If we go still further back, personal insecurity, in the dawn of the last century, or in 1781, when Horace Walpole wrote his amusing stories to the Countess of Ossory, or even in the early youth-time of persons now living, was proverbial. We must, at least, acknowledge that we can now ride and walk in comparative safety.

But this comparative state of social security is not, we fear, so much owing to the moral culture of the universal mind (Robert Owen's parallelograms are not yet established), as to the difficulty of perpetration, in consequence of the improvement in our police. The Bow-street runners may have been active and cunning bull-dogs of the law, but the watchmen were a mere phalanx of old women out of petticoats; and even the mounted dragoon often failed to subdue a riot by the *caltrops* that were strewn along the road.

Even in the late threatened outbreak in the manufacturing districts, such destructive instruments were extensively forged—of these we saw specimens. Therefore, with bad roads, and a woful deficiency of lamps and defenders, we wonder not at the criminal triumphs of Abershaw, Barrington, and Turpin.

The isolated cruelties of the present day prove a latent malevolence still brooding in dens and alleys—witness the sudden ebullition and onslaught of the red republicans of Gaul, that only await the breath of rebellion to light it again to a flame. We ardently hope, nay, confidently believe, this breath will not readily be excited, notwithstanding the bad feeling engendered in the heart by a portion of the current literature of the day.

True, we hear little now of marauders, freebooters, and caterans: a freer intercourse and open roads, and even the footways of the tourist, have revealed passes and fastnesses in Scotland and Cumberland, once only known to the Roys of former days.

But there is one paramount psychical force that is now by steam and post working its gigantic blessings on the world. Facility of intercourse is daily amalgamating the national, the universal mind, and fraternizing the beings of our earth; and doubtless those rulers, who would even now have been guilty of the heinous crime of lawless invasion, have learned, or been impelled by this facility, so to know and to esteem their neighbours, that they have at once sheathed the sword which would constantly have been drawn, and been wet with the blood of an enemy.

Now there is no doubt that bad training is one of the main predisposing causes of crime. "Train up a child in the way he should go, and when he is old he will not depart from it," and "the child is father of the man," are sacred and profane truisms on every one's lips. The evidence of very careful observers, cited in Mr. Hill's third report, is most deplorable, and proves how much of degraded profligacy exists, both in those parents who, having debased themselves, and lost all self-respect, daily neglect and destroy the minds of their offspring, and in those who, falling under the impulse of passion, have produced beings (the natural children of most unnatural parents) either to starve, or live by crime. We therefore coincide with our author that the parent might very fairly be made in a degree responsible for the crimes of his child while under age, just as he is for the contraction of a debt. The producing of a child that *must commit a crime to live*, is an infliction on society that demands the most condign punishment; for it must follow that the field of the mind not cultivated with healthful blossoms will run wild with weeds. A creature of reason that thinks and feels *must*, by the centrifugal force of that intellect, vent and direct it somewhere or to something; and it would indeed be almost a miracle if this *instinctive* being, fraught with impulses and passions homologous with those of the brute, should not indulge and feed them by every slavish mode, for indeed he scarce knows better—he is but a step above the beast of the field. But he has a soul to be saved or lost, and, indeed, lost he *must* be, if not protected; for, like Ishmael, every hand will be raised against him. Now we were almost about to write something like an absolution for these unhappy creatures. It is a delicate ground, we own, to tread on; and yet, taught as we are that where much is given much is required, may we not also hope that for those whose little of good is filched from them, mercy will temper justice in the final award. We scarce know a deeper object of sympathy than such a being as Mr. Barclay has so graphically described, in his pamphlet on "Juvenile Delinquency."

To ensure a happy result—to obviate crime—the culture must be commenced at the dawn of its development by the mother, for, "just as the twig is bent the tree's inclined." We know there is a supreme delight in the little heart of a child who is early taught to read. We do not mean a wearing of the brain with a *course* of study. The *rough hewing* of the great model of mankind, Alfred, was begun by his mother, almost in his nursery; and it is a fine eulogy, even on *his* memory, to record that it was a psychical influence, a literary reward, that was his first stimulus to good.

In this way the blood is *calmly* directed to the tuition and development of the noblest organs—those of intellect—and not exclusively to the sensual. But we must yet economize, and even here ensure a due supply of blood to the organs of assimilation, or we shall mar instead of make; and instead of sending rich and fertile blood to the intellect, it will be a poor and impoverished fluid. The success of Guggenbuhl will prove our position, who ever improves the health of structure in the cretin ere he essays the cultivation of the mind of low standard.

That even in Britain this course of psychical culture is not essentially costly, is shown in the reports of Sheriff Watson, of Aberdeen, Mr. Davies, and others.

We may observe, also, that the cheapness of the posting-rate will tend much to the improvement of the working classes, who are extremely proud of their faculty of correspondence by letter with their friends.

On the subject of "hereditary crime," as it is termed, to which our author draws attention, we must observe that this consists merely in a tendency or predisposition, just as in struma or gout the seeds or germs may be latent even for a life, if due care be observed to keep healthy the crasis of the blood. So, if the moral and intellectual organs be brought into due and healthful play, the hereditary tendency to crime may be readily controlled and thwarted. Psychical as well as physical actions may be equally illustrative of John Hunter's axiom.

Of course there are exceptions where there is permanent disorganization, or preponderance, or deficiency; these cases are of course irremediable, but they are rare. Phrenology may decide that there may thus be an entailment of a disposition to commit crime almost or completely irresistible.

But this monomaniacal disposition to commit crime is not merely hereditary, it is too constantly imparted, and this more especially in the paramount incentive, intoxication, that exhibits in some prisons, as, for instance, that of New York, a percentage over other causes of nearly four-fifths. In the case of Mobbs, executed in November last, drinking was the provocation, both in the murderer and his victim.

The confirmed drunkard is often (for a time) a *furious* madman—that is, during the stimulation of the alcohol: when that has subsided, he becomes the hypochondriac—the *melancholy* madman—and must *repeat* the vice, to lift him out of the slough of despair, which is insupportable. Thus the drinking monomania, like many other minor vices, "grows by what it feeds on." It is a deep psychological subject, but we can here only hint that over-stimulation, by slow and stealthy degrees, wears out the sensibility of the brain and nervous system.

But it does not always require *deep* drinking to constitute a sinner of this class. The sensibility of the brain may in some be so hyper-acute as to be excited to frenzy even by a small quantity of stimulant. The contrasted degrees of this sensibility are wonderful. We knew two clergymen—one really amiable—so constituted, in whom two glasses of wine lighted up the brain so intensely, that the grossest expressions then fell from their lips. On the contrary, a stalwart and hard-working drayman has been known to swallow from ten to twelve pots of porter daily, without intoxication.

The state of the drunkard is a sad decadence of human nature: the alcoholic excitement may not only induce to crime, but it may render the mind and conscience perfectly callous and reckless, so as to destroy all shame, and fear of consequences. The thought of the drunkard is often a selfish and isolated elysium.

Poverty and drunkenness almost invariably go hand in hand. We learn in Mr. Hill's appendix, that many waste in drink 36*s*. out of their earnings of 40*s*., a very large portion, probably, of the sixty-five millions annually spent in Britain on alcoholic fluids.

We were gratified to learn from our author with what facility and impunity the slave of the gin-palace can break through his evil habit. We have the evidence of "an intelligent prisoner," in one of the reports, that "the craving for drink generally dies away in the course of eight or ten days." In the report for 1850, he quotes the affirmation of Mr. Fox, of Derby, that "in 27 years he never knew an injury to health by the sudden withdrawing of stimulating liquors." Of course he does not allude to the condition of delirium tremens.

That the minds of paupers are often willing to break this evil habit the author proves, by their *voluntary* application for admission to a prison, "*to be cured of drinking.*"

We believe that the association of drunkenness with crime forms one of the most profound subjects for the consideration of the psychologist and legislator. (The question of the abolition of the gin-palace, and the injustice of quashing the *vested interest* of a landlord, is foreign to our present criticism.) If we assimilate, we were about to write identify, intoxication with insanity, the resolution of the question would go far to remove one of the dilemmas of the criminal court, or the lunatic commission. May we hazard this proposition. If *slavish* and continued drunkenness be indulged in, and murder be the consequence, the man-slayer is, *in a moral sense*, equally criminal as if he were at the time sober. Paley, with his fine-spun sophistry, argues that a drunken murderer is responsible only for three-fourths of the guilt of a sober one!

Now it is clear that ere the delivery of a verdict on a drunken criminal, it should be inquired, did the murderer *make his own madness by drink?* The degree of homicide by a *self-created* drunkard cannot be far short of that of a sane or sober man; the crime, surely, cannot then be chance medley, or even manslaughter, but murder, and should be *most severely* punished. (We argue not here for or against the *capital*

infliction.) So that the special plea of monomania, that often acquits a man on all other points sane, should, we think, in this instance fail, especially if the culprit be proved to have threatened revenge, and nursed his malevolence against his victim in his *sane or sober moments.*

It is curious to note the influence of an antagonizing passion or force in the control or suspending of crime. The author informs us, in his report for 1847, "that although there were (at that time) about 1000 depositors in the savings-bank at Jedburgh, only one of these depositors, during a period of five years, had been committed to prison" —not merely, we believe, because they had money to spend, but that they felt a sort of pride at being known as people of property and character.

The slightest change of subject for thought or contemplation will often, by the force of novelty, eject for a time a more degraded passion. The author records an extreme diminution of intoxication in a county town, during the week of a travelling exhibition.

In discussing the further means of diminishing poverty and crime, Mr. Hill refers to "the habit of self-control and forethought." But now, even if they could or would listen to the precepts of a devout minister, the callosities of the mind might not, *therefore*, be softened down: for there is often a poison in the brain which requires an antidote more material and potent than moral suasion, which may have, to use a chemical phrase, a greater affinity for the organ of thought than the vice itself: and, indeed, we observe that allusions to such antidotes peep out almost unconsciously throughout the pages of Mr. Hill's book.

Poverty is doubtless a very frequent incentive to crime, but the word poverty itself is a mere *relative* expression. Contentment may be witnessed where there is merely enough to support life, if *comparison* be not brought into the poor man's train of thought. Directly he *compares his want* with *others' luxury*, and especially if he be excited by the imitative monomania of combination, a sort of insurrection is uppermost in his mind, and he is at once for pulling down others to raise himself. Thus the wildfire of the brain blazes out into rebellion, not because others are happier, but more wealthy or honoured than themselves. Perhaps this involves the secret of the psychical influence resulting from a "*direct pecuniary interest*" of the workman in the factory of his employer. "I should augur," writes Mr. Hill, at page 127, "the best results from the plan being carried into general operation, as I believe it would benefit both parties, remove many mistaken and exaggerated ideas on the part of the workmen as to the extent of their employers' gains, increase the sympathies between the different classes of society," &c.

As the minds of men have become enlightened, the penal statutes have been doubtless much improved. The ordeals of burning ploughshares, ear-lopping, ducking-stools, and other ordeals of inquisitorial torture, have been abolished; partly subsequent to the jurisdiction of Sir Matthew Hale, but chiefly since the bill of Sir Samuel Romilly.

The principle of punishment is little understood. It is not based on a system of retaliation or revenge, like the code of the Hindoo and other barbarous or superstitious nations; not exactly in the words of Paley, "the retribution of so much pain for so much guilt," but as a warning and example to the mass. The system of *private* execution, so energetically recommended in the letters of Charles Dickens, is, we think, a mistake. If the public mind be not deterred from crime by the public execution of a criminal, why is it? Partly from the maudlin sympathy and petting lavished on many a capital felon by a set of whimpering sentimentalists, but chiefly because it is believed, and almost proved, that the culprit rarely *suffers physically*. The sensation of hanging, like that of drowning, is rarely a very painful one; and this is very generally known and discussed in the coterie of felons. In proof of this, Mr. Hill refers (p. 173) to a mock trial in a prison described by a felon, "when one of the prisoners sitting as judge, some others acting as witnesses, and others as counsel, all the proceedings of the court of justice were gone through, the sentence pronounced, and mockingly carried into execution. I shall not forget that day when one of these murderers was placed in the cell amongst us beneath the assize court, a few moments after the doom of death had been passed upon him. Coolly pointing the fore-finger of his right hand to his neck, he said, 'I am to hang!'"

As to the sense of degradation and shame in these sinners, it is a term not often found in their vocabulary. On all accounts, then, and especially from its psychical evil, we would not advocate capital punishment; but the penalty for crime should consist in a graduated and properly apportioned infliction of *pain*, for without such infliction of pain—physical or mental—there could be no punishment. Let us not be libelled with the word *Draconian* for this opinion: the penalty we advocate is not an inquisitorial torture to extort from the lips of innocence, but a meet suffering for a proved or confessed crime; but, we repeat, there is no *need* of *death*. But where extreme cruelty has deeply blackened the commission of crime, as in the cases of Tawell, Greenacre, and Rush, what degree of punishment can be termed severity, always supposing the criminal were *aware* of the penalty adjudged to the crime by law? For such, we were almost about to suggest the code of that island king, who said "the *least* punishment we have is death."

Death, indeed, in the midst of sin, is ever an awful end; and the dread expectation is a thousandfold increased when it is the exit of such consummate monsters. To insure the full psychical influence of punishment, there should be a graduated and repeated infliction of *pain*—perpetual, and occasionally silent, imprisonment under scientific surveillance—at least, the public should be taught, or convinced, that it is so. Were this witnessed, then we believe the mob would constantly dread the commission of the crime that called for it. And surely, the most morbid sensibility could not deem even the infliction of mere pain on a thousand cruel, if it were proved to have been the preventive of *one* murder. And then a jury would never be deterred from delivering a verdict of guilty, even on proof of capital crime, if they were sure the culprit's blood would not be on their heads. Pages of nonsense have been written against the disgrace of branding or affixing a permanent mark on a culprit; but we believe the possibility of such a disgrace would be a very efficient prevention of many a deep crime. A criminal who, by his or her heroic bearing on the scaffold, gained the admiration of a mob, would come down from his stilts at once if he were seen to wince under a cat, or carried about the scar of a *brand-iron* on his forehead. We should not then be disgusted with the debased vanity of a Hocker, who, to prepare himself for the scaffold, was engrossed with his toilet when he ought to have been poring over the precepts of his Bible; or the Satanic pride of a Manning in *dying game*, according to the slang phrase, on the scaffold.

All this, we believe, would be merciful—its result, a voluminous saving of life—and, indeed, eventually really abridge suffering in the mass. We should then, at least, hear no more of *suggestions* or temptations to crime by the exhibition of a capital punishment. We believe this mode of punishment would have met all the arguments of Montesquieu, Rousseau, Beccaria, and others who have commented on the insufficiency of our penal laws. With all our apparent severity, we may here record our belief, that the psychological study of many a culprit might often indicate such a state of stricken conscience and deep remorse as to prompt us to implore a pardon; but this should not be granted until the strictest investigation has confirmed the work of contrite repentance. Pardon is sometimes a greater reformer than punishment. The abolition of penal death would, in this also, be a great blessing; it would afford the best chance, even after extreme severity had been endured by a criminal, of psychical amendment. He might be turned into a course even of honesty and virtue. Our prisons would thus be converted from torture-cells into *maisons de santé* of the mind. Mr. Hill comments with much sagacity on this point.* Above

* Page 150.

all, the life of innocence could not then be sacrificed to the uncertainty and chicanery of the law of evidence. Nothing can compensate the family of an innocent victim; but the lavishment of kindness and assistance might go far to compensate a man for the memory of bars and fetters. We quote the following passages from page 151:—

"While it is still left to the courts of justice to determine on the guilt or innocence of the accused, and on the necessity of their withdrawal from society, it may be assigned to those entrusted more or less directly with the reformatory treatment to determine the time of release; subject, however, to a most competent, well-appointed, careful, and responsible supervision and control, and subject to the proviso, that no amount of subsequent good conduct should be considered sufficient to warrant the liberation of a person who had once been guilty of deliberate murder."

Coinciding with much of this, we must yet observe that it may involve a serious error in thoughtless minds, drawing as it does too little distinction between consciousness and responsibility, and their contrasts. The term *criminal lunatic* is a misnomer that has to this day involved much useless discussion and dilemma. Now, seeing that our lunatic asyla are constituted hospitals for cure, and not as in former, indeed very recent days, places for detention, we still think, with Mr. Charles Pearson, that there are cases of monomania, as that of M'Naughten, which might most profitably, and even healthfully, be submitted to a sort of unconscious punishment in the shape of labour. Scientifically apportioned, it would indeed be salutary food both for body and mind; it would concentrate the thought on useful matter of fact, dislodge the burrowing of melancholy, prevent the moroseness of congestion, thus defending the insane from themselves and their dark thoughts, and becoming perhaps the penal, though merciful, preventive of further delinquency.

On the adoption of such a system, however, the inspector must not be a Crown lawyer, but a psycho-physiologist—able to discriminate between malingering and truth. *He* would also be able to decide on the moral or physical convalescence of a prisoner ere he be liberated and permitted again to be at large. Among other modes of effecting parallel improvements, Mr. Hill suggests "to give the judges an unlimited power of imprisonment in certain cases, with a view to assign such period of imprisonment as would be long enough to afford all reasonable opportunity for reformation, the pardoning power of the Crown being exercised whenever it should be *deemed safe* to release the offender before his allotted time."

In allusion to the contrast of this, the protracted detention, we read—

"Let it be observed that in Switzerland and America, where freedom is held as dear as in this country, imprisonment is sometimes awarded for the whole life, and without, therefore, the limit provided by the plan under consideration—a limit depending on cure; and that it is of frequent occurrence that, even as it regards the young and comparatively innocent, an offender is placed in a reformatory school (which is, in fact, a prison), there to be detained, should it be judged necessary, until he be of age, a period generally sufficient to allow of an effective training to habits which will prevent a recurrence to crime."

With all our penal infliction, it is not impossible to combine reformation and cure both of body and mind; and the psychological changes often observed from casual events, sanction and encourage the adoption of a systematic plan.

We must not here widely dilate on the *kind* of punishment most efficient, but we may so far coincide with Mr. Hill as to believe a judicious system of imprisonment more efficacious than transportation—a course never adopted in Belgium, America, or Switzerland. The abolition of transportation was, we believe, hinted at in the last Speech from the Throne, and our colonists in many quarters of the globe entertain a decided objection to it. As our late inspector would regulate it, imprisonment might be far less costly; by judicious management, indeed, almost self-supporting. It would certainly be a safer and surer mode of custody. When amendment—the psychical cure of the propensity—is effected, then, and not till then, may the prisoner be transferred to another clime, not as a convict, but an emigrant.

The separate and silent systems of imprisonment seem most judicious in theory, and so would they be in practice if carefully watched and graduated. Even the prospect of *re-association*, to one enduring the severe penalty of seclusion, will act as a healthy stimulus to the vascular and nervous systems. Anything that induces hope, is effective both in physics and in morals.

The author offers objections to the silent system; but there are some objections to *all* penal inflictions and their execution. An executioner can no more be esteemed than the capital punishment approved. It is said to be *unnatural;* all human punishment is unnatural, since all are equal in God's eye.

*We* really approve of a modified and temporary silent system; and the prisoner, if he think he deserves it, will be patient under its infliction. It preserves him from bad counsel; for all intercourse and converse of criminals usually tend to evil. It gives him time, too, to hold communion with his own heart. This must, of course, be graduated and strictly watched, or imbecility, or even insanity, *may be* the result. The mind, like the body, if *starved,* will die.

The consciousness of error is the first step to repentance, and silence and solitude will often induce reflection, and tend to this result. Like all potent remedies, solitude may be carried too far; but this is no argument against moderate doses. Moderate doses of opium induce healthy slumber; but it should not be decried because an over-dose will kill. The physician should watch the influence of silence and solitude just as he would the effect of bleeding and digitalis in acute inflammation, and desist from both when the mind has been chastened or enough blood has been lost.

It does not appear that silence and separation are so destructive, from the continuation of the stringent system at La Roquette, in Paris. Of its rules we quote the following :—

"Every boy has a separate cell, leaving it only to take exercise in his turn.

"The exercise-yards, chapel, school-room, &c., are all so contrived as to enable the teacher to see and communicate with all the boys at once, but to prevent the boys from seeing each other," &c.

By this adoption we may soon be able to discriminate between the criminal and insane offender—a psychologist would easily make an accurate diagnosis.

Isolated cases of apparent failure must not invalidate this potent mode of correction. Perhaps it may be rejected as too costly; but a rigid economy should be enforced. In Newgate, each prisoner costs nearly 40*l.*; in Bridewell, 50*l.* per annum. The expense of none need extend to 20*l.* per annum.

That separation is useful, we are certain—more, perhaps, in the adult than the juvenile. We think, too, that during association a sort of secondary punishment might be adopted for bad speech or counsel, and thus we might establish a sort of purification in criminal society.

The psychology of the criminal court is one of the most curious subjects for contemplation in the arena of the law; it becomes painfully so when we become conscious of the untruth and misrepresentation which its arguments and its proceedings exhibit. Indeed, if a stranger were to witness the defence of many a culprit for the first time, he would scarce believe, amidst the ribaldry, and, sometimes, utter lack of solemnity, that the life of a human creature was at stake: he would, at another time, blush for the long robe, when he sees "the hall of justice degraded, as it too often is, into a kind of mental boxing ground, where witnesses are insulted and brow-beaten, and where the prisoner, to his surprise, sometimes finds that any acts of trickery and deception which he may have practised (and which probably led to his being then on his trial) are outdone by the well-dressed gentlemen around him, in their power of twisting evidence, distorting facts, and implying, with well-feigned simplicity, the truth of that which they know to be false."—p. 167.

It may be all very fine to admire the sophistry and special pleading of a defence, but the power of advocacy, by making "the worse appear the better reason," is often dangerous in proportion to its power. In the case of Courvoisier, indeed, not only was the guilty on the point of escaping, but an innocent was nearly being arraigned, and might have suffered.

The sacredness of truth, however Samuel Johnson might have counselled Boswell in sanction of the argument to *disprove guilt*, shoul l be far more devoutly observed; and although we would all rather a thousand culprits should escape than that one innocent should be done to death, yet, when evidence is clear and convincing, that a felon should slip his neck out of the noose on the strength of that disgusting quibble, a *flaw in the indictment*, is a palpable disgrace to the criminal law.

"Judex damnatur cum nocens absolvitur."

Now, if in lieu of death we established incarceration for life, unquestionably the degrees of prison discipline should be justly varied and adapted. The cell of a capital convict should be a penal dungeon —a room within dark and unadorned walls, not like those of Holloway and Reading; the culprit should not be proud of the style of his fine mansion. This graduated discipline, especially with the principle of *responsible* inspection now adopted, would complete the abolition of promiscuous association, on which Mr. Hill writes very judiciously. Indeed, the old prisons may well have been termed *seminaries for crime*, especially when scarcely conscious and ignorant youth came under the blighting influence of manhood accomplished in the principle and practice of crime. On the soft and excitable brain of a youth the influence is dazzling and almost electric. Imitation also in the young is half-instinctive; and the boy is at once fascinated by the glowing colours which a Nestor in crime flings over the picture of his career, and he may become at once his slavish admirer, his half-worshipping proselyte.

"To send a child," writes our author, "seven or eight years old to an ordinary prison, to a fortress with grated doors and barred windows, guarded at all points and surrounded by high walls, would seem, when stated in plain terms, to be an act both of folly and cruelty. And when not only the child is treated without regard to the feelings and fears of infancy, but owing to the bad state of the prison, the little thing is placed in a position in which he is in danger of being corrupted for life, the picture in all its features becomes painful and revolting."—p. 158.

From the statistical reports of committals, we regret to learn that it is in the heyday or prime of life that the great mass of crime is committed. It may be, by the unreflecting, presumed that high health may then predominate. But psychology at once discovers *undue* excitement, of the brain especially: the eccentric principle of the will takes a wrong course. But even this erethysm, when *rightly directed*, may aim at the accomplishment of noble deeds; we then term it enthusiasm. But here, alas! the excess of energy expends itself in crime.

There is then often no time for the influence of a moral remedy, but the physician may do much by subduing this excess—this hyperæmia of the cerebro-spinal system. We agree, therefore, that incarceration should be *penal*, and not mere confinement. The prison should be far less comfortable than the union, or we may have some difficulty in *ejecting* a prisoner after his term is completed. The author relates an amusing case, indeed where ejectment was only effected by *smoking* the prisoner *out* of his cell.

The plan which has been advocated, of the participation of a criminal in the profits of his work, may change at once his psychical character. His mind is elevated by the consciousness of his now being useful: he is in fact, so far, *converted*. The author quotes many proofs of the efficacy of this system in the prisons of Munich, New Jersey, &c., and it is asserted that "very few prisoners who earned money under this rule ever returned to prison.". Then, and only then, when he is conscious of this utility, can a prisoner with safety be discharged. It seems, from the authority of Mr. Crawford and Mr. Russell, that the tread-mill is worse than useless. In corroboration, we quote a sentence of Lord Derby when Secretary of the Colonies. "No man ever performs strenuously a task imposed with no other object than that of keeping him employed."

Now, the hygeine of crime is in all this even of deep importance. If we well regard the psychical phases of a case ere we adjudicate upon it, we may often discover that the depravity has its root in monomania; it needs then the infirmary, and not the cell.

A sudden and violent act in a person previously rational, or merely reserved, may indeed be the very outbreak of mania that had been incubating in the brain. It is the hot fit of a fever, after the reverie, the cold stage or rigor, has passed. But even now the mania may be nipped in the bud, and the brain functions preserved, or rather restored, perhaps by one bleeding. Can we forget the case of the statesman who was seen by his physician to attempt to stanch the blood in his neck wound, after its flow had relieved his congested and maddened brain?

We witness illustrations by analogy of the hygeine of crime, in the criminals who labour at agriculture in the open field. "It is well known," writes Mr. Hill, "that there are very few attempts to escape from the well-conducted juvenile prison at Mettray, near Tours; and such attempts are rare also at the prison at Berne, used for adults as well as for children, where the greatest criminals in the canton are confined, and where a large portion of the inmates are employed in agriculture and gardening." This interesting fact, we think, can only be reconcilable on the principle of the hygeine of crime. The brain partakes of the general health of the body, which the tillage and turning up of fresh earth, and the fresh air induce, and the mind feels at once a zest and relief in the occupation. Gil Blas refers to the digging of Count D'Olivarez as a relief to his state of melancholy.

There is hence a joyousness imparted, and the boys, we know, at Parkhurst, at Mettray, and at Red-Hill, scarcely wish for a change. In no other way can we explain why criminals with no cordons around them do not often attempt to escape, however they may fear a *greater* punishment if they are retaken. Perhaps the sedative effect of *fatigue* may in some degree explain this. At Mettray, one great principle is that the boys be thoroughly occupied and thoroughly fatigued. We doubt not, on the contrary, that the garrets and cellars, by inducing disease, engender crime. Scrofula and other asthenic states thus developed, at once predispose to illness and vice. By curing these diseases, a prisoner may be sometimes converted even before his arraignment; and if he were then dismissed, he might at once become a useful member of society. We will quote our lawyer's opinion on this point.

> "If to the early rising, regular employment, cleanliness, proper temperature, good ventilation, and sufficient and wholesome diet, were superadded plenty of work in the open field, the indulgences, under proper regulations, of the natural desire for companionship and the stimulus of hope, I am convinced that a high degree of physical health might be attained, and that the moral health of the prisoners would also be promoted."—p. 262.

Breathing the carbonic acid of so many pulmonary systems invariably reduces the mind's energy; and a change in the current of air may in a moment change the current of thought. The bracing air on the mountain top will, we know, fling a *couleur de rose* on all around, creating, indeed, a sort of mental elysium, far more healthful than the fumes of opium.

If we contrast the habits of industry with those of sloth and sluggardism, which passes in bed perhaps fifteen hours out of the twenty-four, we perceive the woeful difference. John Howard's words are, "Make men diligent, and they will be honest."

From the sympathies between the cerebro-spinal system especially, and the digestive and assimilating organs, it follows that the psychical condition is, to a certain degree, dependent on dietetic rules; and we believe that the economical will be proved the most salutary, as it is the most penal.

In one of the slightest sensibility, the stomach, during the anxieties and doubts of incarceration and future penalties, can bear and dispose properly of little: the ingesta should therefore be light, and also occasionally *changed*, for organization, like the intellect, requires novelty. A long repetition of the same food will occasion loathing, and consequent debility. Oatmeal porridge may, however, still form the staple article of diet. The peasants of Scotland take little else, and we have proved its agreeableness as well as high nutrient property in children especially nurtured in Scotland. According to the assurance of Mr. Hill, the individual dietetic cost per day is 3*d.* The constituent part and properties, indeed, of farinacea, are close upon those of animal fibre. We may add that the Irish labourer, even under heavy work, often takes little more than potatoes and water. Probably the explanation of this will be that he is, psychically considered, a mere animal, and all his vascular and nervous energy goes to alimentary organization.

Almost synchronously with the conversion of lunatic asylums into psychical hospitals, we have, as we have hinted, the conversion of prisons into schools. It is the most important psychological adoption of the age.

The prisoner must, we think, at once feel the superiority of learning to read over the labour of the treadmill. In most it will increase self-respect, while both devout and secular instruction may be instilled imperceptibly into the mind, still, however, forming a modified punishment.

Mr. Hill approves of constituting prisoners tutors and monitors over others. We fear, however, that in this respect the wish for salutary results may often be father to the thought! The Rev. Mr. Russell affirms that "crime is but a matter of instruction:" and the fear that crime is so prone to steal out almost imperceptibly, would persuade us from the recommendation or adoption of such a mode, except under restricted and very inquisitorial regulations.

In *young* minds, doubtless, the *pride of progress* is excited very early, and if this stimulus take the right course, a boy may not only like his school, but become ultimately useful. We take pleasure in quoting an anecdote from Sheriff Watson, the distinguished founder of the schools of Aberdeen, on this point.

"The schools are all in a flourishing condition, the attendance regular, the work and education satisfactory, and the discipline perfect. The other day, when the doctor was making his last visit to a child who had had fever, he found her in tears; on asking what was the matter, the mother replied, 'Indeed, doctor, she is breaking her heart to get back to school, and you must let her return to-morrow.'"

Among the lowest, even in the ragged schools (and we have just now witnessed such a truth), the ambition of being *high up* is an immense stimulus; the most ardent desire is constantly evinced to answer a question first.

Regarding the mode of *religious* instruction in chapel, the author quotes the following evidence of Mr. Reynolds in favour of *open* pews. We repeat it, as it forms an interesting evidence of a long train of serious thought coursing through the mind, even if that had been characterized by obduracy:

"I am happy to state that the removal of the stalls during the past year has been attended by no injurious consequences, while it appears to have answered all the good ends for which the removal had been considered desirable. The prisoner now feels that he is in a house of prayer, engaged in social worship, treated with reasonable confidence, and permitted to hear the offers of divine mercy, without galling marks of his degradation being continually presented to his eye."

The effect of this public religious instruction may be much enhanced by voluntary lay visitation, and exposition of scripture. But we perceive that the testimony even of chaplains is decidedly in favour of blending entertaining literature from a well selected library with the sacred and devout. To these salutary modes may be added the periodical visits of good and exemplary relations, and the habit of correspondence by letter. Of this prisoners are often very proud; and even in the lowly the occupation absorbs for a time the whole thought, so that Satan "finds no mischief still for *idle* hands to do."

All this will go far to induce cheerfulness *from a proper source:* a condition which so much aids the improvement of mind as well as body, for a "merry heart is the life of the flesh."

The effect of wise and judicious government requires, of course, no advocacy: it will be expected that the mind and heart will thereby become soothed and encouraged, and *attachment* even constantly induced. As some proof of this, we will quote from our author the expressions both of a recreant and faithful mind.

"Some prisoners appear to suffer more from the sense of shame in again encountering, after recommitment, a governor who has been kind to them, than from any other part of their punishment. 'All his care has been thrown away,' 'He'll have no hope of me,' are not unfrequent expressions."—p. 308.

"In a letter from a liberated prisoner who is now doing well, the writer, after grateful expressions to many of the officers for instruction in sewing, reading, &c., which she had received in prison, and for the good advice given to her, said, 'Indeed, I was treated with love and respect, which made me think on the evil of my ways, and resolve and endeavour, by God's grace, to renounce them."—p. 310.

On the contrary—

"If the prisoner has been subdued merely by fear, and by a force not addressed to his *reason*, the probability is that on the pressure being withdrawn even for a short time, he will resume his old practices (though probably not on the same spot), and that with a fresh spirit of hostility and recklessness. So, also, if he has been treated, though not with harshness, yet like a child in leading-strings, without any cultivation of the powers of self-control, and still less those of virtuous self action, although he may conduct himself in an exemplary manner in prison, and leave with a sincere desire to live honestly and respectably, he will be so wanting in the power to provide for himself, and to resist temptation, as probably soon again to fall into crime."—p. 285.

Now, if this sort of academic imprisonment be so beneficial to the adult, how much more will it be to the impressible organization of youth. It is far easier "to teach the young idea how to shoot" than the old.

"The success up to this time of the juvenile prison at Mettray has already been mentioned. Great success, also, appears to have attended several institutions in America, which, although called 'houses of refuge,' seem to be, in fact, juvenile prisons. A large proportion (more than two-thirds) of the children who are sent to the juvenile refuge called the Kanke Haus, near Hamburgh, are found to live honestly after they leave, as is the case with the majority of children at the Industrial school at Aberdeen. Again, the large number of boys at the prison in Glasgow, who were found to do well after liberation, under but a slight amount of superintendence kindly extended to them by some Sunday-school teachers, and the fact that of fifty young persons to whom, in the course of two years, the governor of that prison had advanced a little money, some tools, and the materials for labour, forty-eight paid back the whole that had been lent them, as mentioned in my seventh and eighth reports, show how much, under moderately favourable circumstances, may be hoped for from the young."—p. 325.

The Christian spirit that first suggested and established the *farm* system, *fermes hospices*, was Pestalozzi, of Zurich, and the blessings of this early training form a most interesting psychological truth, that a penal institution, paradoxical as it may appear, may form under judgment a school of morals. From his work on "National Education" the author quotes this passage:—

"The first practical knowledge inculcated on a noviciate" (at Hackney-Wick) "is, that his comforts in life will depend mainly on his own exertions; nay, if he indulges in idleness, he may want the very *necessaries* of life. He is informed at the onset that he will have to labour to earn at least a part of his maintenance, before he will have food to eat. Few even of the dullest can be proof against the demonstration." —p. 331.

These elements of success are the principles of the Philanthropic School at Red Hill, so ably superintended by Mr. Sydney Turner, which institution, indeed, formed the model of many of the regulations since adopted at Mettray. It is a pure psychological principle. The boys are "*put on their mettle*," an honest pride and ambition are excited—it is a system of *moral* reward and punishment. We have heard of children who have grumbled out that "it is of no *use* to be good." Such callous and heartless sentiments are not uttered at Red Hill.

In this and other similar institutions, *tickets of credit* are preserved and cherished as a valuable treasure; and we believe the recording angel is far more busy than the accusing spirit even in the memoranda of the "Black Book." The reverend superintendent at Red Hill says:

"This record gives to their actions a sort of perpetuity, the idea of which operates with wonderful force as an incentive to a laudable, and a preventive of an improper conduct. Those who would despise a flogging are kept in awe by the black book (as the calendar of faults is named); and this simple mean has already produced an astonishing effect in the manner of these children, and almost removed every trace of their former evil propensities."—Mettray, p. 23.

Thus is established the immense superiority of psychical influence over mere bodily or *somatic* feeling; and indeed, it almost sanctions the adoption of a brand as a more potent infliction than the gallows. Yet we still acknowledge that, with the callous and obdurate, perhaps the only inducement to good behaviour in a prison, is the dread of *something worse.* Thus, at Mettray, a prison with less stringent rules, one of the chief sources of obedience is the dread of being sent to La Roquette, in Paris, where a dark cell and semi-starvation stare the criminal inmates in the face.

We believe we have thus fairly analyzed our author, and expressed an *epitome* of our sentiments on this important subject. The deep investigation, accurate observation, and practical comments of Mr. Hill, are most valuable. We express our firm belief that he has laid the foundation for a very great amount of improvement in the construction of the criminal code.

